# Development of a 23-Gene Signature for Tumor Growth Mechanism in Vestibular Schwannoma

**DOI:** 10.3390/cancers16244134

**Published:** 2024-12-11

**Authors:** Ji-Yong Sung, Jung Woo Lee

**Affiliations:** 1 Department of Research & Development, VeraOmics, 138 Seoksanro, Namdong-gu, Incheon 21551, Republic of Korea; 2 Department of Biological Sciences, College of Natural Sciences, Seoul National University, Seoul 08826, Republic of Korea; 3 Department of Orthopaedic Surgery, Yonsei University Wonju College of Medicine, Wonju 26493, Republic of Korea; 4 Yonsei Institute of Sports Science and Exercise Medicine, Wonju 26426, Republic of Korea; 5 Biobytes Co., Ltd., Chuncheon 24341, Republic of Korea

**Keywords:** tumor growth signature, vestibular schwannoma, Schwann cell, microglia, 23 signature genes, benign tumor

## Abstract

**Background/Objectives**: Transcriptome profiling can reveal prognostic biomarkers and therapeutic vulnerabilities for directing clinical care. Currently, there are no biomarkers that can accurately predict patient prognosis regarding tumor growth and the tumor immune microenvironment in vestibular schwannomas. This study aimed to investigate the mechanisms of tumor growth using bulk RNA-seq and single-cell data from patients with vestibular schwannomas. **Methods**: Gene set variation analysis was used to assess groups with high and low tumor growth using four cohorts of bulk RNA-seq data (210 patients with vestibular schwannoma), 33,081 single cells, and 558 tumor growth-related genes. **Results**: SIG558, a tumor growth signature gene, was enriched in Schwann cells and microglial cells with high stemness, according to stemness analysis and cell–cell communication analysis of 33,081 single cells. We identified 391 genes that were strongly expressed in Schwann cells with high stemness. In addition, we identified 23 genes related to signal transduction that are important for tumor growth through cell–cell interactions in seven cell types at the single-cell level. **Conclusions**: Our research demonstrates that the 23 signature genes are potential predictors and prognostic biomarkers for direct medical therapy in patients with vestibular schwannoma, and that they should be prospectively verified using large patient cohorts. These results could potentially be used in precision medicine to develop treatment strategies for vestibular schwannomas by targeting these 23 genes.

## Simple Summary

Schwannomas develop from Schwann cells, which are surrounding the nerve fibers, but there are currently no reliable ways to predict how they will grow or affect patients. In this study, we investigated the mechanisms behind tumor growth using patient samples. We analyzed genetic data from both large tumor samples and individual cells to identify genes that influence how the tumor develops. The research discovered 23 specific genes that play a critical role in tumor growth, many of which are linked to key cells in the tumor. These findings are important because they suggest potential biomarkers, or indicators, that could help doctors predict how tumors will behave and choose the best treatment options for patients. This study also lays the groundwork for future research into developing personalized treatments for vestibular schwannomas, with the hope of improving patient outcomes through targeted therapies.

## 1. Introduction

Vestibular schwannomas (VS) are benign tumors that develop from Schwann cells surrounding the eighth cranial nerve. Unlike malignant tumors, benign tumors do not invade or metastasize to surrounding tissues. However, little is known about the characteristics of benign tumors. In VS, as the tumor grows and its size increases, the patient is at risk of hearing loss. If complete tumor removal is difficult, hearing can be improved by reducing tumor growth [1,2]. However, only a few studies have investigated the growth and spread of this tumor.

Neurofibromatosis type 2 (NF2), also known as NF2-related schwannomatosis [3], is primarily caused by loss of function and loss-of-function mutations in the NF2 gene [4]. Several computational cancer genomic studies have used omics data in VS [5,6,7]; however, studies on the mechanisms underlying tumor growth are still lacking. Recently, several cancer genomic studies have suggested strategies for targeting Schwann cells [6]. For example, the MEK–ERK pathway has been successfully targeted in fusion-positive Schwann cells, indicating a potential treatment strategy for this particular tumor population [8]. One study showed that tumor-specific deletions and large structural variants are associated with different tumor growth rates [9].

Vascular endothelial growth factor (VEGF) showed a favorable correlation with tumor development rate in a study of 27 patients. Moreover, several variables have been explored that may affect the growth rate of VS [10]. The patterns of apoptosis, cellular response to hypoxia, and PI3K–Akt, AMPK, FOXO, and chemokine signaling pathways are considerably enriched in the cystic development of VS [11]. In VS, consumption of acetylsalicylic acid has shown no effect on COX2 expression, which is linked to VS tumor expansion and proliferation [12]. Certain genetic, epigenetic, and actionable transcriptional programs, such as the PIGF, VEGF, MEK, and mTOR pathways, have been linked to painful schwannomatosis-related schwannomas and may be targeted to control this illness [13].

Some results suggest that the increase in VS size is not primarily due to Schwann cell growth but also due to the infiltration of macrophages [14]. Such mechanistic studies have enabled tumor targeting; however, they require large-scale data analyses across numerous cohorts.

In this study, we used four cohorts of bulk transcriptome and single-cell data from 33,081 cells to investigate the mechanism of VS growth from a global perspective. We identified 23 signature genes associated with this mechanism. These findings provide important insights into patient stratification, treatment, and drug development from the perspective of precision medicine.

## 2. Methods and Materials

### 2.1. Bulk Transcriptome Analysis

For the meta-analysis, we downloaded the transcriptome profiles of four cohorts (GSE141801, GSE 108524, GSE54934, and GSE39645) of 210 patients with VS using the Gene Expression Omnibus (GEO). Through a literature review of previously published studies, 558 genes related to VS growth were identified [15]. A bulk gene expression profile sample was classified as a single-patient classifier using the gene set variation analysis (GSVA) R package [16]. If the signature gene expression was higher than the average value, it was classified into the high growth signature (GS) group, and if it was lower than the average value, it was classified into the low GS group. Metascape [17] was used for 558 gene ontology analyses, and patient survival analysis was performed for 558 signature genes in the TCGA Pan-Cancer Atlas using GEPIA2 [18]. Gene enrichment was analyzed using cancer hallmark pathway [19] genes from MSigDB (https://www.gsea-msigdb.org/gsea/msigdb/, accessed on 10 August 2023), and related genes from previous studies were used for telomere maintenance mechanism analysis [20,21,22]. CIBERSORT [23] was used for the deconvolution analysis of immune cells, and related genes were imported from the literature for oncogene, tumor suppressor, and stemness analyses. We used 83 KEGG metabolic pathways. Genes related to antigen-presenting cells, nuclear chromosome segregation, and hearing loss were used in MSigDB, and the TIDE algorithm [24] was used to predict the immune checkpoint inhibitor response and cancer-associated fibroblast analysis.

### 2.2. Single-Cell RNA-Seq Analysis

We obtained single-cell data from three patients with VS via email from the authors of a previously published paper [6]. Single-cell UMAP analysis was performed under the default conditions of the Seurat R package [25], and imputation was performed using ALRA [26]. We used the cell types described in previously published papers.

The stemness of each cell type was calculated using the StemID algorithm [27], and the GSVA algorithm [16] for calculating the SIG558 score was run more than 1,000,000 times to increase accuracy. We performed cell–cell communication analysis using the CellChat algorithm [28] and used the Consensus Path DB (http://cpdb.molgen.mpg.de/, accessed on 10 August 2023) for protein–protein interaction analysis [29].

## 3. Results

### 3.1. Hallmark Landscape of VS

**Figure 1:**
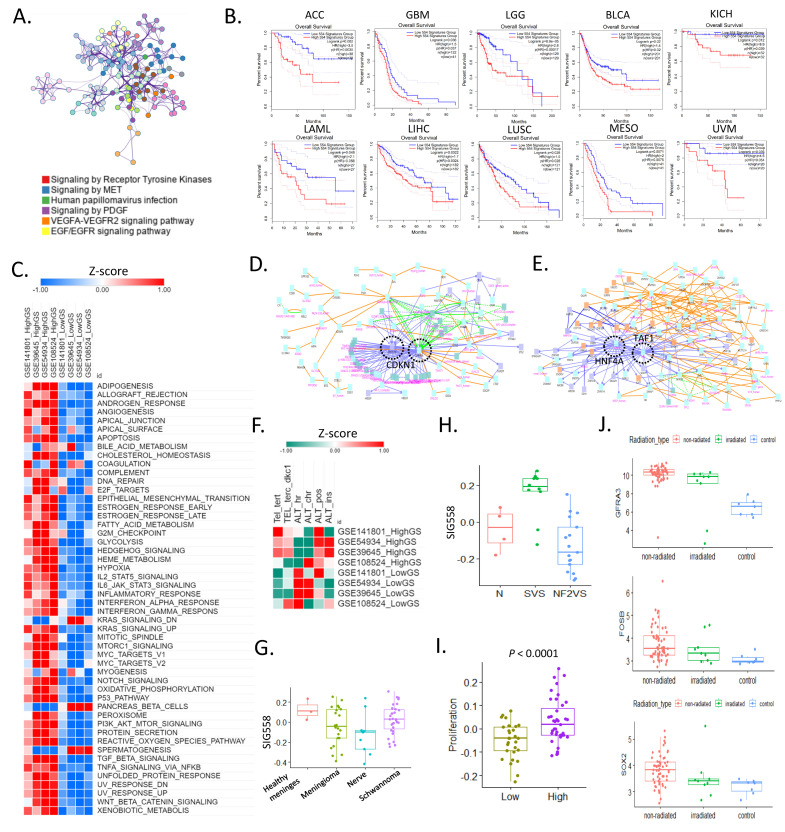
Cancer hallmark landscape of vestibular schwannoma. (**A**) Enriched biological pathways for SIG558 gene ontology. (**B**) Kaplan–Meier plots showing the overall survival rates for the high and low SIG558 groups. The *p*-value was calculated using the log-rank test. The following 10 cancer types had significantly different prognoses: ACC, adrenocortical carcinoma; GBM, glioblastoma multiforme; LGG, brain lower-grade glioma; BLCA, bladder urothelial carcinoma; KICH, kidney chromophobe; LAML, acute myeloid leukemia; LIHC, liver hepatocellular carcinoma; LUSC, lung squamous cell carcinoma; MESO, mesothelioma; UVM, uveal melanoma. (**C**) Heat map of cancer hallmarks between high and low growth groups. (**D**) Protein–protein interaction network for highly expressed genes in the high growth group. (**E**) Protein–protein interaction network for highly expressed genes in the low growth group. (**F**) Heat map of telomere maintenance mechanisms in the high and low growth groups. (**G**) Box plot of enriched SIG558 in four different types of cases. (**H**) Box plot of enriched SIG573 for three different statuses: N, normal nerve; SVS, sporadic vestibular schwannoma; NF2SV, NF2-associated vestibular schwannoma. (**I**) Box plot of proliferation in the high and low growth groups. (**J**) Box plot for radiation therapy-affected genes (*GFRA3*, *FOSB*, *SOX2*).

Our study revealed the target genes and mechanisms of tumor growth in VS using four cohorts of bulk transcriptome and single-cell RNA-seq data. We selected 558 signature genes (Supplementary Table S1) related to VS tumor growth through a literature review. These GS genes were mainly enriched in receptor tyrosine kinase signaling, MET signaling, human papillomavirus infection, PDGF signaling, the VEGFA–VEGFR2 signaling pathway, and the EGF–EGFR signaling pathway (Figure 1A). We analyzed 33 TCGA cancer types to determine the relationship between these genes and patient prognosis in different cancer types. Groups with high expression of these genes showed poor prognoses in 10 cancer types: adrenocortical carcinoma, glioblastoma multiforme (GBM), brain lower-grade glioma (LGG), bladder urothelial carcinoma, kidney chromophobe, acute myeloid leukemia, liver hepatocellular carcinoma, lung squamous cell carcinoma, mesothelioma, and uveal melanoma (Figure 1B). In contrast, in kidney clear cell carcinoma, the high expression group of SIG558 showed a good prognosis (Supplementary Figure S1). We classified the bulk RNA-seq samples into high and low GS groups using the GSVA algorithm in four bulk RNA-seq cohorts and compared the cancer hallmarks. In the high GS group, the activities of most cancer hallmark pathways were high. However, KRAS signaling was downregulated, and pancreatic beta cells and spermatogenesis showed high activity in the low GS group. (Figure 1C).

We analyzed the gene regulation network and protein–protein interactions based on differentially expressed genes in the high and low GS groups. CDKN1A was linked to the hub protein in the high GS group, and HNF4A and TAF1 were the hub proteins in the low GS group (Figure 1D,E). Overall, we analyzed six pathways related to telomere maintenance in four cohorts of 210 patients, and telomerase and alternative lengthening of telomere (ALT) activities were high in the high GS group. In contrast, in the low GS group, telomerase activity was low or absent, and ALT activity was high (Figure 1F). The low GS group exhibited ALT-like tumor characteristics. Telomere maintenance mechanisms are involved in tumor immortalization, and ALT is an alternative mechanism used in the absence of telomerase [21].

In addition, when SIG558 was analyzed in patients with various types of low-grade meningiomas and schwannomas, it showed the lowest activity in the nerves and lower activity in meningiomas than in healthy meninges (Figure 1G). In addition, we investigated how SIG558 expression differed among various schwannoma types. SIG558 showed significantly higher activity in sporadic VS and the lowest activity in NF2-associated VS (Figure 1H). A significantly higher proliferation rate was observed in the high GS group (Figure 1I). In addition, we discovered that three out of eleven schwannoma marker genes differed according to radiation therapy. Compared with the non-irradiated group, the irradiated group showed decreased expression levels of *GFRA3*, *FOSB*, and *SOX2* (Figure 1J). These results revealed the characteristics of high GS tumors, along with the hallmarks of VS.

### 3.2. Tumor Immune Microenvironment of Tumor Growth in VS

The tumor immune microenvironment reprograms tumors in a direction favorable for survival. In particular, metabolic reprogramming occurs when tumors use energy to compete for nutrients with other cells, and the tumor immune microenvironment provides strong indications of tumor characteristics.

**Figure 2:**
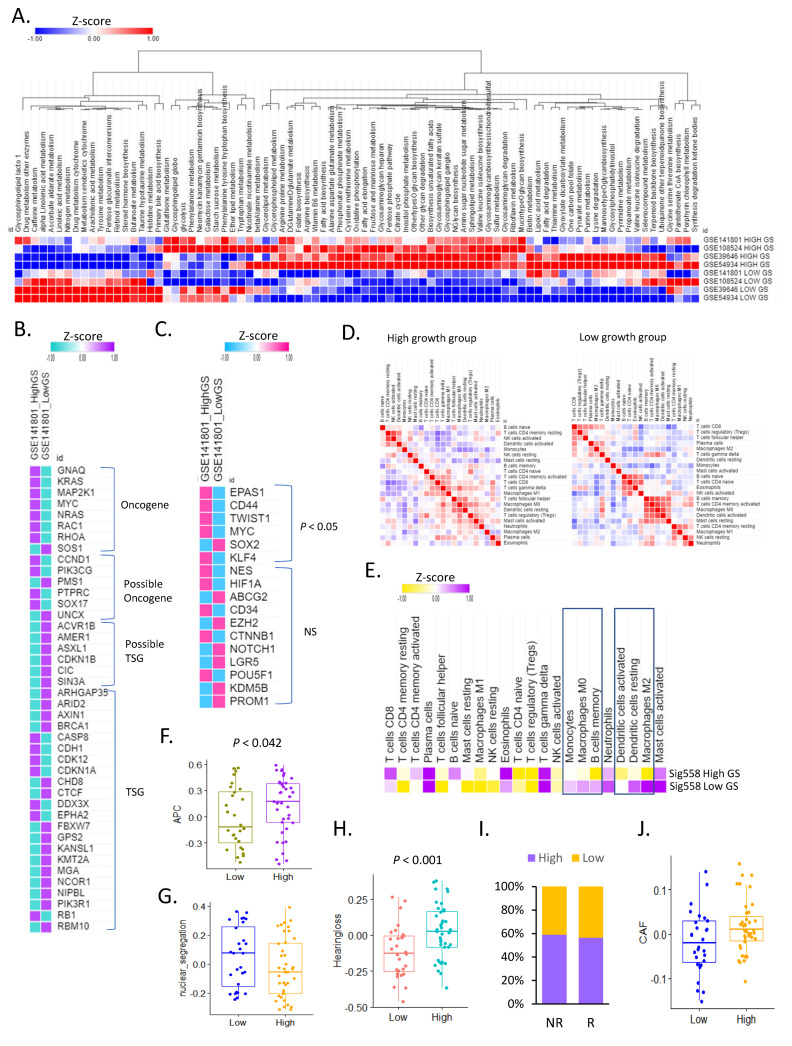
Tumor immune microenvironment of tumor growth in vestibular schwannoma. (**A**) Heat map of metabolic pathway activity between the high and low growth groups in four cohorts. (**B**) Heat map of oncogenes and tumor suppressor genes (TSG) in high and low growth groups. (**C**) Heat map of stemness-related genes in the high and low growth groups. (**D**) Similarity matrix of immune cell types between the high and low growth groups. (**E**) Heat map of the correlation between SIG558 and immune cell types in the high and low growth groups. (**F**) Box plot of antigen-presenting cell activity in the high and low growth groups. (**G**) Box plot of nuclear chromosome segregation activity in the high and low growth groups. (**H**) Box plot for hearing loss activity in the high and low growth groups. (**I**) Bar graph for predicted immune checkpoint inhibitor response in the high and low growth groups. (**J**) Box plot for cancer-associated fibroblast activity in the high and low growth groups.

Through the analysis of 83 KEGG metabolic pathways, we identified the metabolic pathways that differed between the high and low GS groups. Most metabolic pathways showed high activity in the high GS group, and approximately 20% of the metabolic pathways showed high activity in the low GS group (Figure 2A). In addition, when comparing oncogenes and tumor suppressor genes, 85.72% of the oncogenes were highly expressed in the high GS group. In contrast, the expression of most tumor suppressor genes was high in the low GS group, and the expression of *CASP8*, *CDH1*, *CDK12*, *DDX3X*, *EPHA2*, and *RB1* was high in the high GS group (Figure 2B). Among the genes associated with stemness in the high GS group, *EPAS1*, *CD44*, *TWIST1*, *MYC*, *SOX2*, and *KLF4* were significantly upregulated (Figure 2C).

Despite a robust adaptive immune response, the rapid growth of the remaining VS after subtotal surgical resection may be accompanied by T-cell immunosenescence [30]. Using CIBERSORT deconvolution analysis, we found that the immune environment differed between the high and low GS groups. In the high GS group, naïve B cells, resting CD4 memory T cells, activated NK cells, and activated dendritic cells showed similar trends, while CD4 naïve, CD4 memory-activated, and CD8+ T cells also showed similar trends (Figure 2D).

In contrast, in the low GS group, the similarity between CD8 T cells and regulatory T cells was high. These immune cells exhibited different correlations with SIG558 expression. Monocytes, M0 macrophages, memory B cells, activated dendritic cells, resting dendritic cells, and M2 macrophages exhibited different correlations with SIG558 expression. Interestingly, the positive correlation between CD8+ T cells and SIG558 expression was strong in the high GS group (R = 0.8) (Figure 2E). SIG558 and regulatory T cells were negatively correlated, whereas naïve B cells in the high GS group were positively correlated.

To characterize these immune environments, we found that the expression of APC-associated genes was significantly higher in the high GS group (Figure 2F). These characteristics could predict high immune activity in the high tumor growth group. We analyzed the activity of nuclear chromosome segregation-associated genes that can predict aneuploidy in VS compared to ALT tumors, which are associated with a good prognosis in GBM [31]. We found that this activity was significantly higher in the VS of the low GS group (Figure 2G). The expression of 34 genes related to hearing loss was higher in the high GS group (Figure 2H). When predicting the immune checkpoint inhibitor response of the high and low GS groups through the T-cell function of VS, no significant difference was observed between non-responders and responders (Figure 2I). The number of cancer-associated fibroblasts was significantly higher in the high GS group than in the low GS group (Figure 2J).

### 3.3. SIG558 Is Enriched in Schwann and Microglia Cells at the Single-Cell Level

**Figure 3:**
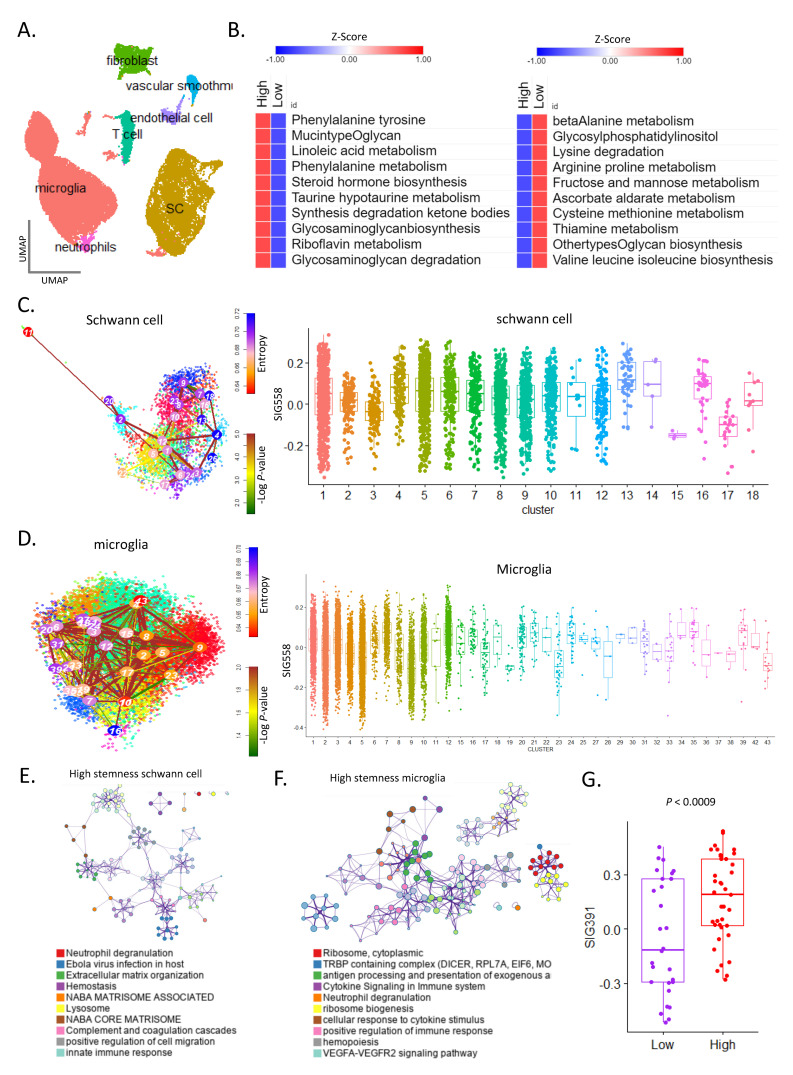
Analysis of cell–cell communication reveals a 24-gene signature for tumor growth mechanism. (**A**) UMAP of seven cell types in vestibular schwannoma of three patients. (**B**) Heat map of the enriched metabolic pathway in the high (**left**) and low (**right**) SIG558 groups. (**C**) tSNE plot of stemness for Schwann cells and box plot of SIG558 for different stem clusters. (**D**) tSNE plot of stemness for microglia cells and box plot of SIG558 for different stem clusters. (**E**) Gene ontology analysis for high-stemness Schwann cells. (**F**) Gene ontology analysis for high-stemness microglia cells. (**G**) Box plot for SIG391 (highly differentially expressed genes in high-stemness Schwann cells).

We performed a single-cell analysis of three patients with VS (Supplementary Figure S2A) and obtained 33,081 cells and 10,032 Schwann cells as tumor cells. The cells were divided into seven types, and the ratio of microglia to Schwann cells was relatively high (Figure 3A). At the single-cell level, we observed the characteristics of Schwann cells, which are tumor cells, and, in particular, the type of metabolic energy used for tumor growth.

The top 10 preferred metabolic pathways were identified in the high and low GS groups. In the high GS group, the phenylalanine tyrosine and mucin-type glycan pathway activities were high, while that of the beta-alanine metabolism and glycosylphosphatidylinositol pathways were high in the low GS group (Figure 3B). Tumor growth differed depending on the metabolic energy preferred by Schwann cells. SIG558 was enriched in Schwann cells (Supplementary Figure S2B. Further, SIG558 was differently enriched depending on the stemness of Schwann cells, and SIG558 activity was confirmed to be exceptionally high in the cluster where the stemness transition occurred within the high-stemness cluster (Figure 3C, Supplementary Figure S3A). In microglia, SIG558 was enriched as stemness increased (Figure 3D, Supplementary Figure S3B). Our analysis confirmed that cells with high stemness exhibited high tumor growth.

Next, we analyzed the gene ontology of genes with high expression in tumor cells with high stemness. Schwann cell signature genes with high stemness were enriched in neutrophil degranulation, extracellular matrix organization, and the NABA core matrix pathway [32] (Figure 3E, Supplementary Table S2). Microglia with high stemness were enriched in the cytoplasmic ribosome, TRBP-containing complex, and VEGFA–VEGFR2 signaling pathway (Figure 3F, Supplementary Table S3). Using bulk RNA-seq data from different VS cohorts, we validated that 391 genes (Supplementary Table S2) that were highly expressed in Schwann cells with high stemness were highly expressed in the high GS group (Figure 3G). We identified that these 391 genes were important in the mechanism related to Schwann cell tumor growth at the single-cell level.

### 3.4. Analysis of Cell–Cell Communication Reveals 23 Signature Genes for the Tumor Growth Mechanism

**Figure 4:**
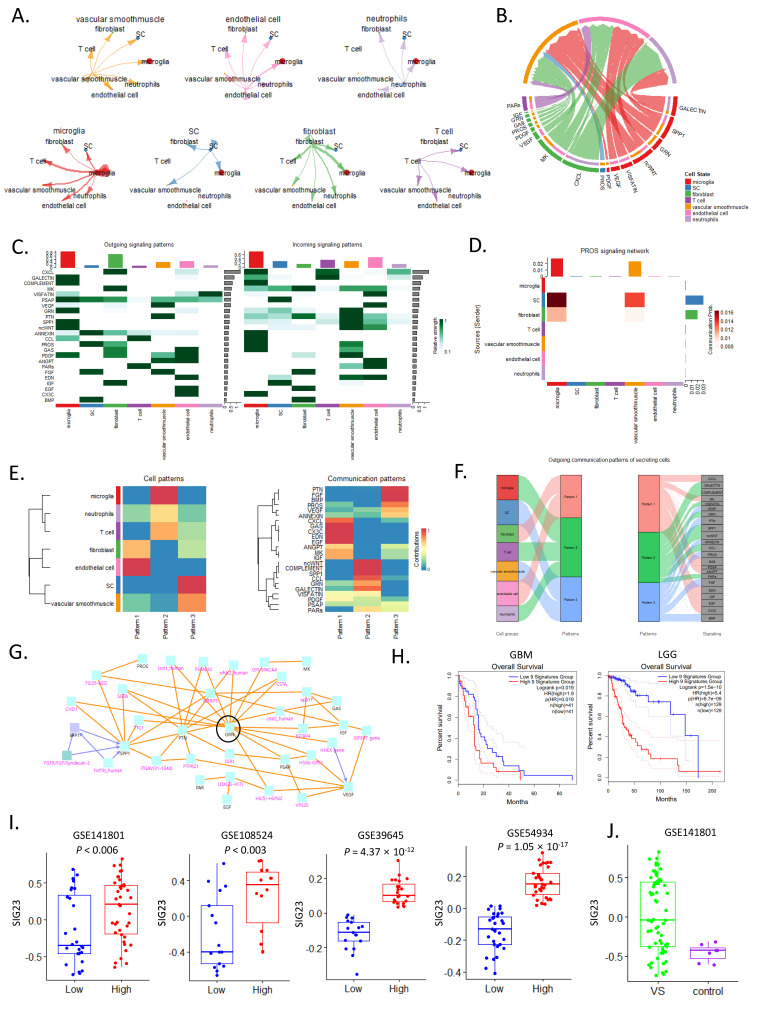
Analysis of inference and cell–cell communication. (**A**) Cell–cell communication network showing the signaling sent from each cell group. (**B**) Chord diagram for the signaling of seven cell types. (**C**) Heat map for the identification of signals contributing the most to outgoing or incoming signaling of certain cell groups. (**D**) Heat map of the PROS signaling network. (**E**) Heat map of cell patterns and communication patterns. (**F**) River plot of outgoing communication patterns of secreting cells. (**G**) Protein–protein interaction network for 23 high tumor growth-related genes. (**H**) Kaplan–Meier plots show the overall survival rates for the high and low SIG9 groups. The *p*-values were analyzed using the log-rank test and adjusted by the Bonferroni correction. (**I**) Box plot of SIG23 in four cohorts. (**J**) Box plot of SIG23 in VS and normal controls.

Analyzing cell-to-cell communication at the single-cell level can be an important approach for understanding tumor growth mechanisms. We analyzed the interactions among the seven cell types using the CellChat algorithm. Microglia interacted the most with other cells, while Schwann cells interacted with three cell types (fibroblasts, vascular smooth muscle cells, and microglia) (Figure 4A,B). The outgoing and incoming signaling patterns were different for each cell type, and the predicted ligand played a role in signal transmission. We defined 24 genes (Supplementary Table S4) as signature genes for VS growth, which functioned in the three communication patterns (Figure 4C). In the PIK3CA-related overgrowth spectrum (PROS) signaling network [33], the communication probability was highest between microglia and Schwann cells, followed by that between Schwann cells and vascular smooth muscle cells (Figure 4D). Cell patterns are largely classified into three types: Schwann cells, vascular smooth muscle cells, fibroblasts, and endothelial cells; neutrophils and T cells; and microglia, classified as neutrophils and T cells. Regarding the communication patterns, GAS, CXCL, EDN, and EGF contributed the most to pattern 1; ncWNT, COMPLEMENT, SPP1, CCLGALECTIN, and GRN contributed the most to pattern 2; and PTN, FGF, BMP, and PROS contributed the most to pattern 3 (Figure 4E).

When analyzing the cell types that contributed to each pattern, Schwann cells and vascular smooth muscle cells were grouped in pattern 3, and in the case of outgoing communication patterns of secreting cells, pattern 3 was linked to VEGF, GRN, PTN, ANNEXIN, PROS, FGF, and BMP signaling (Figure 4F). We analyzed the protein–protein interactions of 23 genes using Consensus Path DB-human and found that the *GRN* gene was connected to many nodes as a hub gene (Figure 4G). We confirmed the prognosis of 23 genes using the GBM and LGG datasets, which represent TCGA brain tumors, and found a poor prognosis in the high GS group (GBM, *p* = 0.019; LGG, *p* = 1.5 × 10^−10^) (Figure 4H). A comparison of the 23 genes between the high and low GS groups in the four bulk transcriptome cohorts confirmed a significant difference between all four cohorts (Figure 4I). Significantly higher levels were observed in the VS group than in the normal control group (Figure 4J). These results indicate a pharmacological target for VS and lay the foundation for a better understanding of the tumor growth mechanisms of VS.

## 4. Discussion

The growth mechanism of VS is well known to be associated with abnormalities in the Merlin protein [34], a tumor suppressor gene that activates receptor tyrosine kinases, such as ErbB, PDGF, VEGF, and C-kit, and activates the downstream pathways PI3K/AKT/mTOR and MEK-ERK1/2. This signal is activated to promote tumor growth and differentiation. In this study, we identified 24 genes at the single-cell level and the mechanisms involved in tumor growth. The VS bulk transcriptome data meta-analysis predicted poor prognosis and hearing loss in the high GS group for the following reasons. First, VS is classified as a brain tumor, and with respect to the telomere maintenance mechanism in VS, telomerase activity was very low in the low GS group. If the low GS group is predicted to be ALT-like, ALT levels in brain tumors can predict a very good prognosis. Second, proliferation was high in the high GS group, and all cancer-related traits showed high activity. Third, although immune function was highly expressed in the high GS group, the prognosis was expected to be good in the low GS group with low immune function. These characteristics closely resemble those observed in GBM ALT tumors. Fourth, the immune checkpoint inhibitor response predicted by the difference in T-cell function was not significantly different between the tumor growth groups. Fifth, the tumor GS gene SIG558 is mainly enriched in Schwann cells and microglial cells with high stemness. We selected 391 highly expressed genes according to the high stemness of Schwann cells (which are tumor cells) and found that they were related to the tumor growth of VS. Sixth, 23 genes were found to play an important role in the tumor growth mechanism—as a result of analyzing the signals exchanged between cells according to the cell type—at the single-cell level. These genes were significantly upregulated in the high GS group. Therefore, we present a precision medicine approach for VS treatment based on the targeting of these 23 genes.

## 5. Conclusions

According to the findings of our study, the 23 signature genes have the potential to serve as predictors and prognostic biomarkers for direct medical therapy in patients with vestibular schwannoma. Furthermore, it is recommended that these genes be prospectively evaluated by employing large patient cohorts. These findings have the potential to be utilized in precision medicine for the purpose of developing therapeutic options for vestibular schwannomas by focusing on these 23 genes.

## Data Availability

The data presented in this study are available in this article and Supplementary Materials.

## Supplementary Materials

The following supporting information can be downloaded from https://www.mdpi.com/article/10.3390/cancers16244134/s1, Figure S1: Kaplan–Meier plots showing the overall survival rates for the high 554 signature group and low 554 signature group; Figure S2: (A) UMAP of three patients (B) Box plot for signature 558 in three cell types; Figure S3: (A) Bar graph for stemness in schwann cells (B) Bar graph for stemness in microglia cells; Table S1: Tumor growth-related gene signature; Table S2: Highly differentially expressed genes in high-stemness tumors (Schwann cells); Table S3: Highly differentially expressed genes in high-stemness microglia cells; Table S4: The 23 gene signature of cell–cell communication for the tumor growth mechanism.

## Abbreviations

ALT, alternative lengthening of telomere; ACC, adrenocortical carcinoma; BLCA, bladder urothelial carcinoma; GBM, glioblastoma multiforme; GS, growth signature; GSVA, gene set variation analysis; KICH, kidney chromophobe; LAML, acute myeloid leukemia; LGG, brain lower-grade glioma; LIHC, liver hepatocellular carcinoma; LUSC, lung squamous cell carcinoma; MESO, mesothelioma; NF2, neurofibromatosis type 2; VEGF, vascular endothelial growth factor; UVM, uveal melanoma; VS, vestibular schwannoma.
