# Supplementary material for: Development of a 23-Gene Signature for Tumor Growth Mechanism in Vestibular Schwannoma
Source: Cancers (Basel). 2024 Dec 11;16(24):4134. doi: 10.3390/cancers16244134 (PMC11674088; doi:10.3390/cancers16244134)
Supplement: Supplementary file 1 [file cancers-16-04134-s001.zip › cancers-3268388-supplementary.docx]

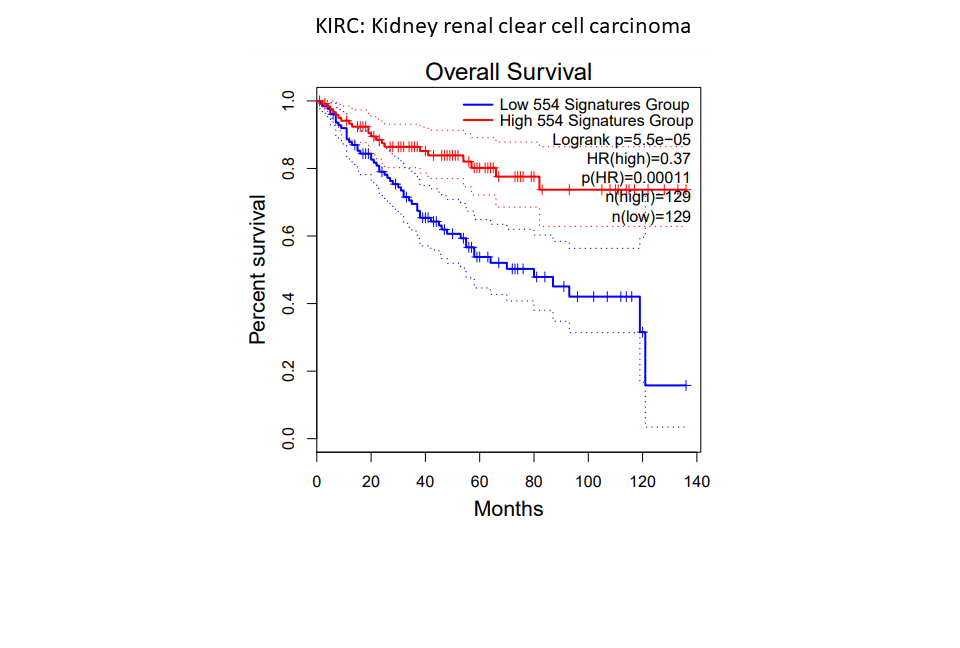


**Figure S1.** Kaplan–Meier plots showing the overall survival rates for the high 554 signature group and low 554 signature group.


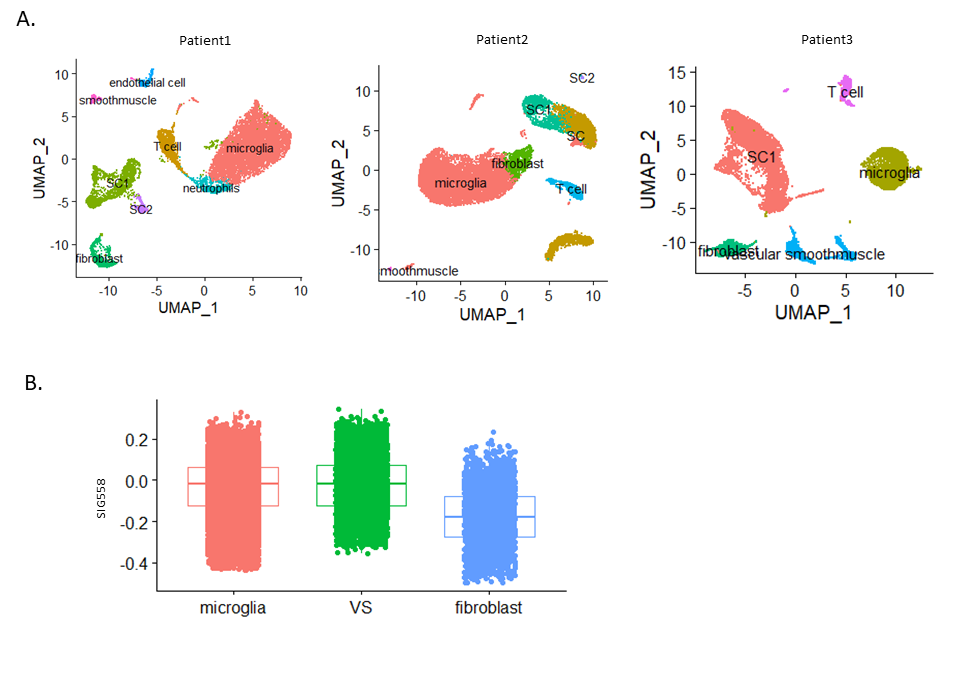


**Figure S2.** (A) UMAP of three patients (B) Box plot for signature 558 in three cell types.


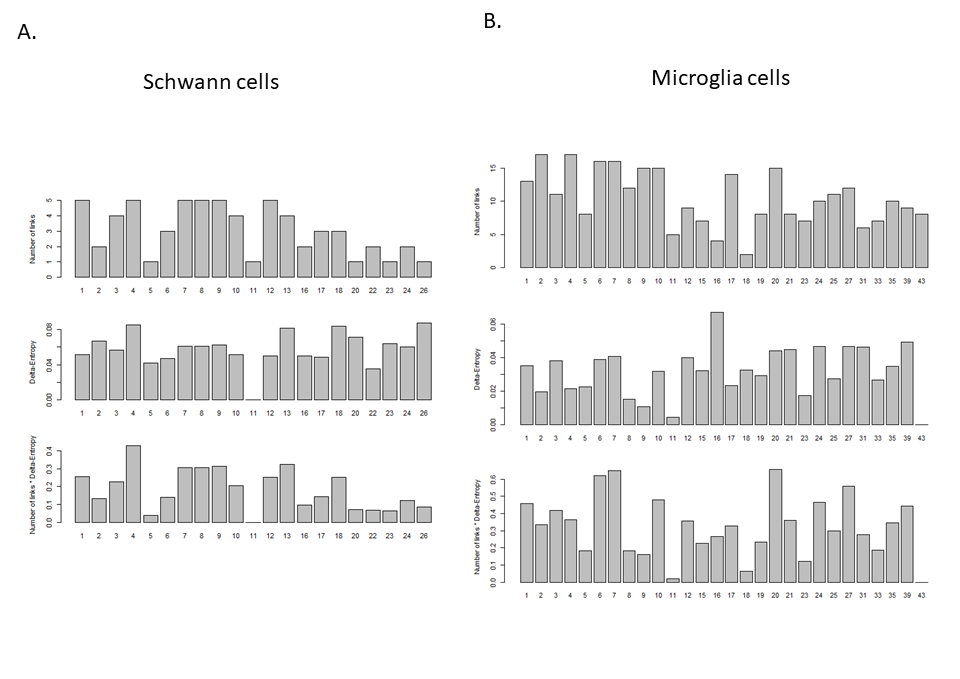


**Figure S3.** (A) Bar graph for stemness in schwann cells (B) Bar graph for stemness in microglia cells.
